# Effect of Salinity on Hydroxylamine Oxidation in a Marine Ammonia-Oxidizing Gammaproteobacterium, *Nitrosococcus oceani* strain NS58: Molecular and Catalytic Properties of Tetraheme Cytochrome *c*-554

**DOI:** 10.1264/jsme2.ME09154e

**Published:** 2014-07-19

Takeshi Hozuki^1^, Tomonori Ohtsuka^1^, Kentaro Arai^1^, Katsuhiko Yoshimatsu^1^, Shigeyasu Tanaka^2^, and Taketomo Fujiwara^1,2,*^

^1^Department of Biological Science, Graduate School of Science, Shizuoka University; and ^2^Integrated Bioscience Section, Graduate School of Science and Technology, Shizuoka University, 836 Oh-ya, Suruga-ku, Shizuoka 422–8529, Japan

Volume 25, no. 2, Page 95–102, 2010

Page 98, column 2, line 28 from the top, ‘The N-terminal amino acid sequence of the protein was determined as IPDELYEALGVDKYKASPKE. The sequence was identical to the deduced sequence of the 31st to 50th residues of the HAO precursor encoded in the noc_0892 gene of the *N. oceani* ATCC19707’ should read ‘The N-terminal amino acid sequence of the protein was determined as DIPDELYEALGVDKYKASPK. The sequence was identical to the deduced sequence of the 30th to 49th residues of the HAO precursor encoded in the noc_0892 gene of the *N. oceani* ATCC19707’.

